# Development, Dynamic Modeling, and Multi-Modal Control of a Therapeutic Exoskeleton for Upper Limb Rehabilitation Training

**DOI:** 10.3390/s18113611

**Published:** 2018-10-24

**Authors:** Qingcong Wu, Hongtao Wu

**Affiliations:** 1 College of Mechanical and Electrical Engineering, Nanjing University of Aeronautics and Astronautics, Nanjing 210016, China; 2 State Key Laboratory of Robotics and System, Harbin Institute of Technology (HIT), Harbin 150001, China

**Keywords:** upper limb exoskeleton, rehabilitation training, multi-modal control, impedance controller, active participation, training intensity

## Abstract

Robot-assisted training is a promising technology in clinical rehabilitation providing effective treatment to the patients with motor disability. In this paper, a multi-modal control strategy for a therapeutic upper limb exoskeleton is proposed to assist the disabled persons perform patient-passive training and patient-cooperative training. A comprehensive overview of the exoskeleton with seven actuated degrees of freedom is introduced. The dynamic modeling and parameters identification strategies of the human-robot interaction system are analyzed. Moreover, an adaptive sliding mode controller with disturbance observer (ASMCDO) is developed to ensure the position control accuracy in patient-passive training. A cascade-proportional-integral-derivative (CPID)-based impedance controller with graphical game-like interface is designed to improve interaction compliance and motivate the active participation of patients in patient-cooperative training. Three typical experiments are conducted to verify the feasibility of the proposed control strategy, including the trajectory tracking experiments, the trajectory tracking experiments with impedance adjustment, and the intention-based training experiments. The experimental results suggest that the tracking error of ASMCDO controller is smaller than that of terminal sliding mode controller. By optimally changing the impedance parameters of CPID-based impedance controller, the training intensity can be adjusted to meet the requirement of different patients.

## 1. Introduction

Over the past decade, the increasing stroke patient population has brought great economic and medical pressures to the whole society. Surviving stroke patients usually have a lower quality of life dues to physical disability and cognitive impairment. Studies on clinical stroke treatment indicate that appropriate rehabilitation training has positive therapeutic effects for avoiding muscle atrophy and recovering musculoskeletal motor functions. However, the conventional one-on-one manual-assisted movement training conducted by physiotherapists suffers from many inherent limitations, such as high labor intensity, high cost, long time consumption, lack of repeatability, low participation levels of patient, and high dependence on personnel with specialized skills [1,2]. In recent years, robot-assisted rehabilitation therapies have gained growing interest from academic researchers and the healthcare industry around the world due to their unique advantages and promising application perspectives. Compared with the traditional manual rehabilitation treatment, the combination of robotic technologies and clinical experience can significantly improve the performance and quality of training. Robot-assisted therapy is capable of delivering high-intensity, long-endurance, goal-directed, and low-cost rehabilitation treatment. Moreover, the functional motivations of patient can be activated to enhance active participation and recover cognitive functions. The physical parameters and therapy data can be recorded and analyzed via sensing system, and that can provide objective basis to optimize training strategy and accelerate recovery process [3,4].

Many therapeutic robot system have been developed to assist stroke patients with motor dysfunctions perform the desired rehabilitation training. The existing rehabilitation robotic devices can be categorized into two types, i.e., end-effector-based robots and exoskeleton-based robots. End-effector-based robot has only a connection between its distal end and the impaired extremity of patient. However, the movement of end-effector cannot uniquely identify the configuration of human limb due to the kinematic redundancy. Miller et al. developed a lightweight and potable end-effector-based therapeutic robot, which is integrated with a wrist and finger force sensor module named WFES, for the upper limb rehabilitation training of hemiplegic stroke patients [5]. Pedro et al. developed a parallel kinematic mechanism (PKM) with two translational and two rotational degrees of freedom (DOFs) for knee diagnosis and rehabilitation tasks [6]. Kang et al. proposed a modular and reconfigurable wrist robot called CR2-Haptic for post-stroke subjects to train forearm and wrist movements [7]. Besides, many other end-effector-based therapeutic robot have been investigated and can be referred to [8,9,10,11,12,13]. Comparatively, the exoskeleton-based rehabilitation robots are developed with more complex structures imitating the anatomical human skeleton and guaranteeing the alignment between the joints axis of robot and impaired limb. ChARMin is a powered exoskeleton integrated with audiovisual game-like interface. It can provide intensive pediatric arm rehabilitation training for the children and adolescents with affected motor functions [14]. Simon et al. proposed a spherical shoulder exoskeleton with a double parallelogram linkage to eliminate singularities and achieve good manipulability properties [15]. Crea et al. developed a semi-autonomous whole-arm exoskeleton for the stroke patients performing activities of daily living (ADL) utilizing hybrid electroencephalography and electrooculography feedback signals [16]. Many other representative exoskeleton-based therapeutic robot have also been designed, such as CAREX-7 [17], RUPERT [18], ULEL [19], ArmeoPower [20], Indego [21], and ETS-MARSE [22].

The effectiveness of robot-assisted rehabilitation training depends on the control strategies applied in the therapeutic robot system. Currently, many kinds of control strategies have been developed according to the requirements of patients with various impairment severities in different therapy periods. The existing control schemes can be basically divided into two categories based on the interaction between therapeutic robots and patients, i.e., patient-passive training control and patient-cooperative training control. During the acute period of hemiplegia, the impaired extremity is fully paralyzed without any muscle contraction. The patient-passive training can imitate the manual therapeutic actions of a physiotherapist. It is especially well suited for the patients with severe paralysis to passively execute repetitive reaching missions along predefined training trajectories. However, it is a challenge to guarantee the position control accuracy during rehabilitation training due to the highly nonlinear properties and unexpected uncertainties of human-robot interaction. Different kinds of control algorithms have been developed to improve control performance of patient-passive training, including neural proportional-integral-derivative (PID) control [23], neural proportional-integral (PI) control [24], adaptive nonsingular terminal sliding mode control (SMC) [25], disturbance observer-based fuzzy control [26], neural-fuzzy adaptive control [27], adaptive backlash compensation control [28], and so on. Comparatively, the patient-cooperation training is applicable for the patients at the comparative recovery period, who have regained parts of motor functions. Clinical studies show that integrating the voluntary efforts of patients into rehabilitation training benefits to accelerate recovery progress and promote psychological confidence. Thus, patient-cooperation training should be able to regulate the human-robot interaction in accordance with the motion intentions and hemiplegia degrees of patients. Many patient-cooperation control strategies have been proposed, such as minimal assist-as-needed controller [29], myoelectric pattern recognition controller [30], electromyography (EMG)-based model predictive controllers [31], subject-adaptive controller [32], and fuzzy adaptive admittance controller [33].

Taking the above into consideration, the contribution of this paper is to develop an upper limb exoskeleton to assist the patient with motor disabilities perform multi-modal rehabilitation training. Firstly, the overall mechanical structure and the MATLAB/xPC-based real-time control system of the proposed therapeutic robot are introduced. Secondly, the dynamic modeling of the human-robot system is researched, and the dynamics parameters are obtained via virtual prototype and calibration experiments. After that, a multi-modal control strategy integrated with an adaptive sliding mode controller and a cascade-proportional-integral-derivative (CPID)-based impedance controller is proposed. The controller is combined with an audiovisual therapy interface and is able to realize patient-passive and patient-cooperation training based on the motor ability of patient. Finally, the effectiveness and feasibility of the developed rehabilitation exoskeleton system and control scheme are verified through three experiments conducted by several volunteers.

## 2. System Description

### 2.1. Exoskeleton Robot Design

**Figure 1:**
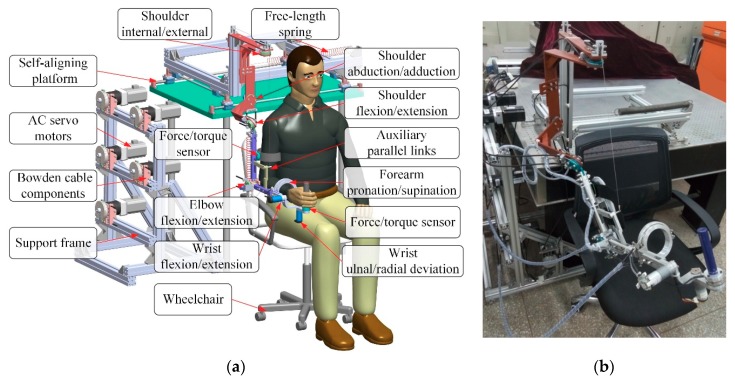
Architecture and major components of the upper extremity rehabilitation exoskeleton. (**a**) Virtual prototype model. (**b**) Real-life picture of exoskeleton.

**Figure 2:**
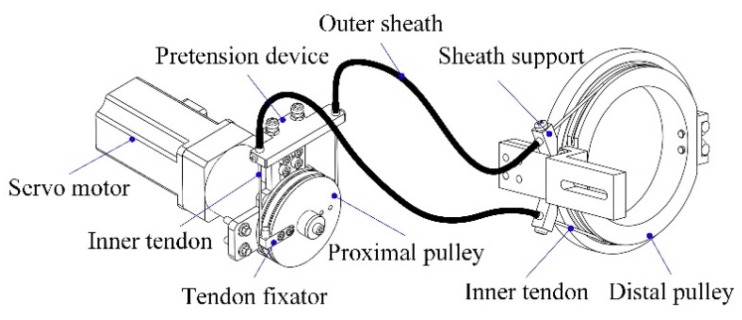
Schematic of the flexible double-tendon-sheath transmission system.

The architecture and major components of the developed upper limb exoskeleton for rehabilitation training are depicted in Figure 1. It has been designed as a wearable interface imitating the anatomy of human skeleton and can be worn on the upper limb of disabled patient via custom Velcro cuffs [34]. The force-feedback exoskeleton robot is characterized by an open-chain kinematics consisting of seven actuated revolute DOFs and two passive translational DOFs covering most of the range of movement (ROM) in ADL. More specifically, there are three revolute DOFs at the shoulder working as a spherical joint for internal/external rotation, abduction/adduction, and flexion/ extension, one revolute joint for elbow flexion/extension, one revolute joint for forearm pronation/supination, and two mutually orthogonal revolute joints at wrist for flexion/extension and ulnal/radial deviation. Since the human shoulder is composed of three DOFs at the glenohumeral joint and two DOFs at the sternoclavicular joint, it is unreasonable to simplify the shoulder as a conventional spherical joint. Thus, the exoskeleton device is mounted at a self-aligning platform with two passive translational DOFs to compensate the misalignment between human shoulder and robot shoulder. The actuated robot joints at shoulder, elbow and forearm are driven by the servo geared motors (SGMAV-04A, reduction ration: 40, YASKAWA Inc., Fukuoka, Japan) located at a fixed support frame remote from the exoskeleton. The driving torques generated in servo motors are transmitted to robot joints via flexible double-tendon-sheath transmission systems, as shown in Figure 2. The inner tendons are attached to the proximal and distal pulleys in pull-pull configuration, while the outer sheathes are fixed to the sheath supports at each side. The pretension device at proximal end is capable of modulating tendon pretension and eliminating undesired slacking problem [35]. Such an arrangement facilitates to reduce the mass, inertial, and volume of the moving parts. The elbow joint is driven by two high precision coreless servo motors (JG-37, ASLONG Inc., Shenzhen, China). A gravity-balanced mechanism consisting of zero free-length springs, pulleys, and auxiliary parallel links is designed and included in the robotic structure to compensate the gravity of upper limb and exoskeleton [36]. The configuration of exoskeleton is measured by the high-precision potentiometers (WDJ22A-50K, OMTER Inc., Shanghai, China) encapsulated into robot joints. The human-robot interaction forces are detected via two six-axis force/torque sensors (NANO-25, ATI, Apex, NC, USA) located at the upper arm and end-effector of exoskeleton, respectively. Most of the exoskeleton components are made of aluminum alloy in order to decrease body weight and enhance mechanical strength. The link lengths of upper arm and forearm, the height of shoulder, as well as the size of each cuff are adjustable, which allows covering the arm size of the wearer with a body height ranging from 1.6 m to 1.9 m. For safety, mechanical stops are mounted on the actuated joints to avoid collisions and excessive motion. Besides, two emergency stop buttons are available for the disabled patient and physical therapist to prevent hazards during rehabilitation training.

### 2.2. Electrical Control System

**Figure 3:**
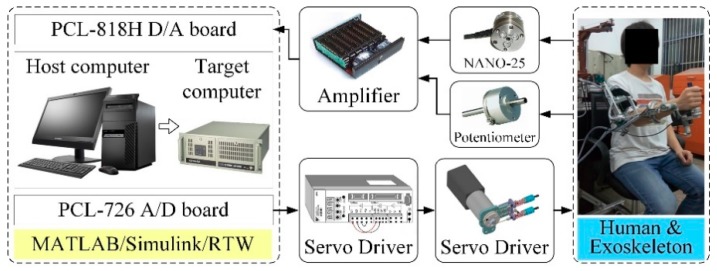
Matlab/Real-Time-Workshop/xPC control system.

The closed-loop control of the therapeutic exoskeleton is executed on a real-time control platform established in MATLAB/xPC environment with the Real-Time-Workshop kernel. A general hardware architecture diagram of the system is depicted in Figure 3. The platform contains two industrial personal computers (IPC-610H, Advantech Inc., Taipei, Taiwan) working as the host computer and target computer respectively. The control programs are firstly developed in the host computer using Simulink software package (Mathworks Inc., Natick, MA, USA). After that, the Simulink models are transformed into executable codes and transmitted to the target computer through RS232 serial port. The analog feedback signals measured by potentiometers and force/torque sensors are acquired via two data acquisition cards (PCL-818, Advantech Inc., Taipei, Taiwan) installed in the target computer. The digital control algorithms are converted into analog output commands via two industrial digital-to-analog cards (PCL-726, Advantech Inc., Taipei, Taiwan), and then delivered to servo drivers to regulate the running of motors. The sampling rate of real-time feedback control and data logging of the platform is set to 1 kHz.

## 3. Dynamics Modeling and Calibration

Generally, the overall dynamic model of the upper limb human-robot interaction system can be formulated by Lagrangian dynamic Equation [37]:

(1) $${M(\theta )\ddot{\theta }+V(\theta ,\dot{\theta })+\tau }_{f}(\theta ,\dot{\theta })+\lambda_{u}-J_{1}^{T}{(\theta )\Gamma }_{1}-J_{2}^{T}{(\theta )\Gamma }_{2}=\tau \;$$

Here, $\theta ,\dot{\theta },\;\mathrm{and}\;\ddot{\theta }\in {\mathbb{R}}^{7}$ are the vectors of the actuated joint position, velocity, and acceleration.$M(\theta )\in {\mathbb{R}}^{7\times 7}$ denotes the symmetric positive definite inertia matrix. $V(\theta ,\dot{\theta })\in {\mathbb{R}}^{7}$ represents the Centripetal and Coriolis torques. $\tau_{f}(\theta ,\dot{\theta })\in {\mathbb{R}}^{7}$ denotes the vector of friction from robot joints, motor reducers, and double-tendon-sheath transmission system. $\lambda_{u}\in {\mathbb{R}}^{7}$ is the lumped effects of uncertainties and modeling errors. $J_{1}^{}(\theta )$ and $J_{2}^{}(\theta )\in {\mathbb{R}}^{6}$ are the Jacobian matrixes providing mapping from the interaction forces at upper arm and end effector to the torques at robot joints. $\Gamma_{1}$ and $\Gamma_{2}\in {\mathbb{R}}^{6}$ are the Cartesian interaction forces measured by force/torque sensors.$\tau \in {\mathbb{R}}^{7}$ denotes the driving torques of servo motors.

It should be noted that the gravitational force is eliminated during dynamic modeling, as the overall system is in gravity-balanced status [36]. Besides, the ROM and velocity of the self-aligning platform is relatively small, thus it is assumed to be stationary for modeling simplification.

The friction model can be expanded as follows:

(2) $$\tau_{f}(\theta ,\dot{\theta })=\tau_{r}(\theta ,\dot{\theta })+\tau_{s}(\theta ,\dot{\theta })\;$$

where $\tau_{r}(\theta ,\dot{\theta })\in {\mathbb{R}}^{7}$ represents the lumped friction from reducers and robot joints; $\tau_{s}(\theta ,\dot{\theta })\in {\mathbb{R}}^{7}$ denotes the friction from double-tendon-sheath transmission system.

Then, the resultant friction torque from reducers and robot joints can be simplified by using the Coulomb-viscous model [38]:

(3) $$\tau_{r,i}=sign({\dot{\theta }}_{i})\tau_{c,i}+b_{i}{\dot{\theta }}_{i}\;$$

Here, *τ_r_*_,*i*_ represent the lumped friction from the *i*th reducer and robot joint. *b_i_* and *τ_c_*_,_*_i_* denotes the corresponding viscous coefficient and Coulomb friction torque.

The torque transmission model of double-tendon-sheath transmission system, which has been developed and verified in our previous research, can be expressed as follows [35]:

(4) $$\psi_{1}\tau_{out,i}=\psi_{2}\tau_{in,i}-sign({\dot{\theta }}_{i})2T_{i}\psi_{1}\psi_{2}\beta_{i}\mu_{i}\;$$

where *τ_in_*_,*i*_ is the input torque of the *i*th proximal pulley. *τ_out_*_,*i*_ represents the output torque acting on the *i*th robot joint. *T_i_*, *β_i_*, and *μ_i_* denote the tendon pretension, accumulated bending angle and Coulomb friction coefficient of the *i*th double-tendon-sheath unit. The radiuses of proximal and distal pulleys are defined as $\psi_{1}$ and $\psi_{2}$, which are all equal to 25 mm in the proposed exoskeleton. *sign*(‧) denotes the sign function

Therefore, the torque transmission model can be rewritten as follow [35]:

(5) $$\tau_{s,i}=\tau_{in,i}-\tau_{out,i}=sign({\dot{\theta }}_{i})2T_{i}\psi_{2}\beta_{i}\mu_{i}\;$$

Here, *τ_s_*_,*i*_ represents the friction torque from the *i*th double-tendon-sheath transmission unit.

Thus, from Equations (2), (3), and (5), the *i*th element of friction vector $\tau_{f}(\theta ,\dot{\theta })$ can be shown as:

(6) $$\tau_{f,i}=sign({\dot{\theta }}_{i})2T_{i}\psi_{2}\beta_{i}\mu_{i}+sign({\dot{\theta }}_{i})\tau_{c,i}+b_{i}{\dot{\theta }}_{i}=sign({\dot{\theta }}_{i})\tau_{e,i}+b_{i}{\dot{\theta }}_{i}\;$$

where *τ_e_*_,*i*_ represents the equivalent coefficient for Coulomb friction torque.

The mass and length of each robot link, the inertia matrix, and the Coriolis/centrifugal torques of the developed dynamic model can be obtained via the computer-aided virtual prototype established in the SolidWorks environment (Dassault Systems, Concord, MA, USA). The detailed model parameters and ROM of each joint are shown in Table 1.

Assuming that the lumped effects of uncertainties of system dynamics model can be eliminated, and the exoskeleton robot is separated from the wearer, then according to Equation (1), the friction torque can be calculated via the following equation:

(7) $$\tau_{f}(\theta ,\dot{\theta })=\tau -M(\theta )\ddot{\theta }-V(\theta ,\dot{\theta })\;$$

Here, the inertia term and the Coriolis/centrifugal term can be obtained via the virtual prototype of exoskeleton. The angular positions of robot joints can be measured by potentiometers, while the velocity and acceleration can be calculated by using the dedicated Kalman filters.

Parameters identification experiments were carried out to achieve accurate system friction models. To find the viscous coefficient and equivalent Coulomb friction torque of Equation (6), the experiments were carried out as the following procedures:

Step (a): The servomotor of the targeted identification joint was set to run in torque control mode and track a sinusoidal trajectory with fixed frequency, while other motors were set to run in braking mode and remain in fixed position.
Step (b): The angular displacement, velocity, acceleration, and driving torque of the targeted identification joint were measured and calculated respectively. The friction torque was computed according to Equation (7).
Step (c): The trajectory tracking experiment was repeated and recorded with different frequency.
Step (d): Based on the relation between angular velocity and friction torque, terms *b_i_* and *τ_e_*_,*i*_ can be identified by employing the least squares fitting method to the experimental data.
Step (e): The targeted identification joint was changed, and the steps from (a) to (d) were repeated to acquire the entire friction model parameters.

**Figure 4:**
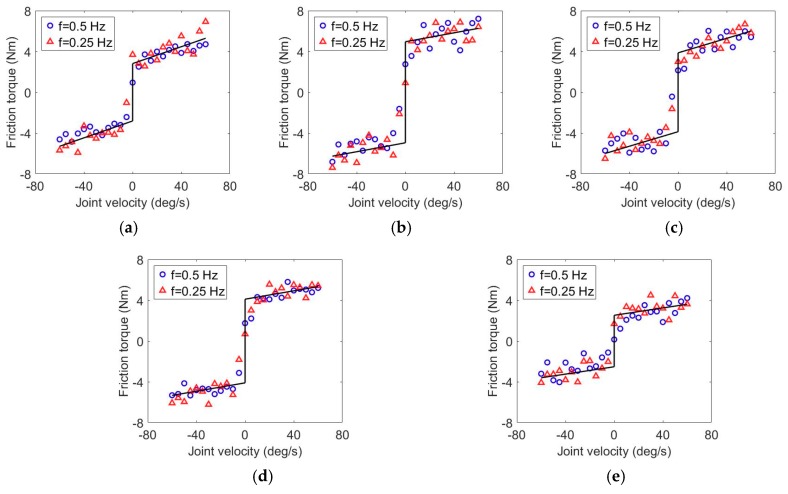
Experimental calibration results of the friction models of exoskeleton joints. (**a**) Shoulder internal/external. (**b**) Shoulder abduction/adduction. (**c**) Shoulder flexion/extension. (**d**) Elbow flexion/extension. (**e**) Forearm pronation/supination.

The flexion/extension and ulnal/radial deviation joints of wrist are directly actuated by motors without Bowden-cable transmission system, joint bearings and external reducer, thus the friction models of these two joints can be obtained by referring to the motor specification and manual. The results of identification experiments with two different frequencies (0.5 Hz and 0.25 Hz) for shoulder internal/external rotation, shoulder abduction/adduction, shoulder flexion/extension, elbow flexion/extension and elbow pronation/supination are presented in Figure 4. The friction torque is plotted against the corresponding joint velocity within the range of −60 deg/s to 60 deg/s. The identified equivalent Coulomb friction coefficient and viscous coefficient, as well as the root mean square errors (RMSE) between the actual frictions and fitting results, are shown in Table 2. 

The RMSE is defined as:$\mathrm{RMSE}=\sqrt{\frac{1}{Q}\sum_{i=1}^{Q}e_{i}^{2}}$, where *e_i_* represents the *i*th error data; *Q* denotes the number of error data.

## 4. Development of Multi-Modular Control Strategy

**Figure 5:**
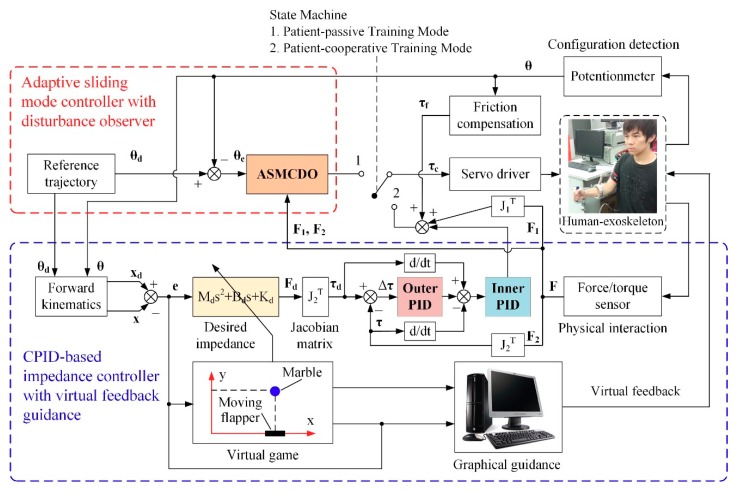
The overall block diagram of the developed multi-modal control strategy.

To satisfy the training requirements of the patients with different weakness levels, a multi-modal control strategy is developed to provide patient-passive training and patient-cooperative training. Firstly, an adaptive sliding mode controller with disturbance observer (ASMCDO) is proposed to realize the patient-passive training mode and prevent joint degeneration. In this training mode, the exoskeleton can move the affected arm of fully paralyzed patient through predetermined trajectories while the patient remains passive. Next, a CPID-based impedance controller integrated with virtual feed guidance is developed, which can assist the patients regaining parts of motor functions perform patient-cooperative training mode and activate their active participation. In this training mode, patients are allowed to actively perform rehabilitation training programs under the visual and acoustical guidance from virtual environment. The interaction characteristics between exoskeleton and affected arm can be quantitatively adjusted via the predefined impedance parameters, while the inner force regulation of impedance control is based on a CPID controller proposed in our previous research [39]. A state machine is integrated into the multi-modal controller to switch different training modes. The overall block diagram of the developed control strategy is shown in Figure 5.

### 4.1. Adaptive Sliding Mode Control with Disturbance Observer

With the aim of reducing the position tracking error of patient-passive mode training in the presence of modeling errors and external uncertainties, an adaptive sliding mode control scheme with a high-order disturbance observer is developed in our research.

The dynamic model of the human-robot interaction system shown in Equation (1) can be rewritten as follows:

(8) $$\ddot{\theta }=M^{-1}(\theta )\left[\tau -V(\theta ,\;\dot{\theta })-\tau_{f}(\theta ,\;\dot{\theta })+J_{1}^{T}(\theta )\Gamma_{1}+J_{2}^{T}(\theta )\Gamma_{2}\right]+\lambda \;$$

Here, $\lambda =-M^{-1}(\theta )\lambda_{u}$ represents the unknown compound disturbance, which is assumed to have a bounded *r*th derivative and satisfies the following equation [40]:

(9) $$\left\|\lambda^{\left(r\right)}\right\|\leq \sigma \;$$

where σ is a positive constant representing the upper bound of unknown disturbance. *λ*^(*r*)^ represents the *r*th derivative of *λ*. Then, a linear disturbance model is utilized to describe the disturbance under assumption (9), which is given by [40]:

(10) $$\left\{ \begin{array}{l} \lambda =D\eta \\ \dot{\eta }=L\lambda^{\left(r\right)}+N\eta \end{array}\right.\;$$

Here, $\eta \in {\mathbb{R}}^{7r}$ is an auxiliary vector. $N\in {\mathbb{R}}^{7r\times 7r}$, $L\in {\mathbb{R}}^{7r\times 7}$, and $D\in {\mathbb{R}}^{7\times 7r}$ denote the system matrices having the following forms:

(11) $$D=\left[\begin{array}{cc} I_{7} & O_{7\times (7r-7)} \end{array}\right],L=\left[\begin{array}{c} O_{(7r-7)\times 7}\\ I_{7} \end{array}\right],N=\left[\begin{array}{cc} O_{(7r-7)\times 7} & I_{7r-7}\\ O_{7\times 7} & O_{7\times (7r-7)} \end{array}\right]\;$$

By combining Equations (8) and (10), an extended system can be obtained as follows:

(12) $$\left\{ \begin{array}{l} \;\ddot{\theta }=M^{-1}(\theta )\left[\tau -V(\theta ,\;\dot{\theta })-\tau_{f}(\theta ,\;\dot{\theta })+J_{1}^{T}(\theta )\Gamma_{1}+J_{2}^{T}(\theta )\Gamma_{2}\right]+D\eta \\ \dot{\eta }=N\eta +L\lambda^{\left(r\right)} \end{array}\right.\;$$

Then, define a nonlinear invertible transformation as follow [40]:

(13) $$\left\{ \begin{array}{l} k_{1}=\ddot{\theta }\\ k_{2}=\eta -g(k_{1}) \end{array}\right.\;$$

Here, $g(k_{1})$ denotes a polynomial vector to be determined [41]. Then, according to Equations (8)–(13), the dynamic of *k*_1_ and *k*_2_ can be obtained:

(14) $$\left\{ \begin{array}{l} {\dot{k}}_{1}=M^{-1}(\theta )\left[\tau -V(\theta ,\;\dot{\theta })-\tau_{f}(\theta ,\;\dot{\theta }){+J}_{1}^{T}(\theta )\Gamma_{1}{+J}_{2}^{T}(\theta )\Gamma_{2}\right]+D\eta \\ {\dot{k}}_{2}=\left[N-\frac{\partial g(k_{1})}{\partial k_{1}}D\right]k_{2}+Ng(k_{1})+L\lambda^{\left(r\right)}-\\ \;\frac{\partial g(k_{1})}{\partial k_{1}}\left\{M^{-1}(\theta )\left[\tau -V(\theta ,\;\dot{\theta })-\tau_{f}(\theta ,\;\dot{\theta }){+J}_{1}^{T}(\theta )\Gamma_{1}{+J}_{2}^{T}(\theta )\Gamma_{2}\right]+Dg(k_{1})\right\} \end{array}\right.\;$$

Then, a high-order observer is proposed to estimate the disturbance as follows [40]:

(15) $$\left\{ \begin{array}{l} {\dot{\hat{k}}}_{2}=Ng(k_{1})+\left[N-\frac{\partial g(k_{1})}{\partial k_{1}}D\right]{\hat{k}}_{2}-\\ \;\frac{\partial g(k_{1})}{\partial k_{1}}\left\{M^{-1}(\theta )\left[\tau -V(\theta ,\;\dot{\theta })-\tau_{f}(\theta ,\;\dot{\theta }){+J}_{1}^{T}(\theta )\Gamma_{1}{+J}_{2}^{T}(\theta )\Gamma_{2}\right]+Dg(k_{1})\right\}\\ \hat{\eta }=g(k_{1})+{\hat{k}}_{2}\\ \hat{\lambda }=D\hat{\eta } \end{array}\right.\;$$

where ${\hat{k}}_{2}$, $\hat{\eta }$, and $\hat{\lambda }$ represent the estimate of $k_{2}$, η, and λ respectively.

The estimation error of auxiliary vector η can be defined as:

(16) $$e_{\eta }=\eta -\hat{\eta }\;$$

Combining Equations (12) and (15) into Equation (16), the derivative of $e_{\eta }$ can be obtained [40]:

(17) $$\begin{array}{l} {\dot{e}}_{\eta }=\dot{\eta }-\dot{\hat{\eta }}=N\eta +L\lambda^{\left(r\right)}-\left[N-\frac{\partial g(k_{1})}{\partial k_{1}}D\right]{\hat{k}}_{2}-Ng(k_{1})-\dot{g}(k_{1})+\\ \;\frac{\partial g(k_{1})}{\partial k_{1}}\left\{M^{-1}(\theta )\left[\tau -V(\theta ,\;\dot{\theta })-\tau_{f}(\theta ,\;\dot{\theta })+J_{1}^{T}(\theta )\Gamma_{1}+J_{2}^{T}(\theta )\Gamma_{2}\right]+Dg(k_{1})\right\}\\ \;=\left[N-\frac{\partial g(k_{1})}{\partial k_{1}}D\right]e_{\eta }+L\lambda^{\left(r\right)} \end{array}$$

It should be pointed out that, if the eigenvalues of $N-\frac{\partial g(k_{1})}{\partial k_{1}}D$ are all located at the left half-plane, the error system shown in Equation (17) will become bounded-input-bounded-output stable, and the disturbance estimation error, which is defined as $\tilde{\lambda }=\lambda -\hat{\lambda }$, is always bounded as:

(18) $$\left\|\tilde{\lambda }\right\|\leq \varphi \;$$

Here, *φ* is a positive constant representing the upper bound of disturbance estimation error.

In order to improve the position tracking performance of the developed exoskeleton in patient-passive rehabilitation training, a sliding control law integrated with a disturbance estimation compensation is developed. The position tracking error is defined as follows:

(19) $$e=\theta_{d}-\theta \;$$

where $e\in {\mathbb{R}}^{7}$ denotes the position tracking error; $\theta_{d}\in {\mathbb{R}}^{7}$ represents the reference tracking trajectory.

The switching function of the sliding mode surface is defined in the tracking error state and written as follows:

(20) $$s=\zeta_{1}e+\zeta_{2}\dot{e}\;$$

where $s\in {\mathbb{R}}^{7}$ denotes the sliding variable vector; $\zeta_{1}\;$ and $\zeta_{2}\in {\mathbb{R}}^{7\times 7}$ represents two positive diagonal matrixes of proportional gains to be designed.

An exponential convergence with a saturation function is used to determine the reaching law of the sliding mode control:

(21) $$\dot{s}=-hs-\alpha sat(s)\;$$

Here, $\alpha \in {\mathbb{R}}^{7\times 7}$ and $h\in {\mathbb{R}}^{7\times 7}$ represent two positive diagonal matrixes; *sat* (‧) denotes the saturation function defined as follows [42]:

(22) $$sat(s)=\left\{ \begin{array}{ll} \frac{s}{\delta }, & \mathrm{when}\;\left|s\right|\leq \delta \\ sign(s), & \mathrm{when}\;\left|s\right|>\delta \end{array}\right.\;$$

where *δ* is a positive constant determining the boundary layer thickness of the saturation function.

Invoking Equations (8), (19), and (20), the derivation of the sliding variable vector with respect to time can be obtained as follows:

(23) $$\begin{array}{l} \dot{s}=\zeta_{1}\dot{e}+\zeta_{2}\ddot{e}\\ =\zeta_{1}\dot{e}+({\ddot{\theta }}_{d}-\ddot{\theta })\\ =\zeta_{1}\dot{e}+\zeta_{2}\left\{{\ddot{\theta }}_{d}-M^{-1}(\theta )\left[\tau -V(\theta ,\;\dot{\theta })-\tau_{f}(\theta ,\dot{\theta })+J_{1}^{T}(\theta )\Gamma_{1}+J_{2}^{T}(\theta )\Gamma_{2}\right]-\lambda \right\} \end{array}$$

Then, insert Equations (15) and (21) into Equation (23), the overall sliding mode controller with disturbance observer can be given as follows:

(24) $$\begin{array}{l} \tau =\frac{M(\theta )}{\zeta_{2}}\left[\zeta_{1}\dot{e}+\zeta_{2}{\ddot{\theta }}_{d}+\alpha sat(s)+hs+\hat{\varphi }sat(s)-\zeta_{2}\hat{\lambda }\right]+\\ \;V(\theta ,\dot{\theta })+\tau_{f}(\theta ,\dot{\theta })-J_{1}^{T}(\theta )\Gamma_{1}-J_{2}^{T}(\theta )\Gamma_{2} \end{array}$$

Here, $\hat{\varphi }$ represents the estimation of upper bound φ, which satisfies the adaptive control law as follows:

(25) $$\dot{\hat{\varphi }}=\upsilon \left\|s\right\|\;$$

where υ is a predefined positive constant.

Then the derivation of sliding variable vector can be rewritten by inserting Equations (24) and (25) into Equation (23), as follows:

(26) $$\dot{s}=-hs-(\alpha +\hat{\varphi })sat(s)-\tilde{\lambda }\;$$

The system stability with the developed controller is demonstrated via the Lyapunov stability theory [27]. Let the Lyapunov function candidate chosen as:

(27) $$V=\frac{1}{2}s^{T}s+\frac{1}{2\upsilon }{\tilde{\varphi }}^{2}\;$$

Here, $\tilde{\varphi }=\varphi -\hat{\varphi }$ denotes the estimation error of φ. Since φ is a constant, we can get:

(28) $$\dot{\tilde{\varphi }}=\dot{\varphi }-\dot{\hat{\varphi }}=-\dot{\hat{\varphi }}\;$$

Differentiating *V* with respect to time and combining Equations (26) and (27), we can obtain that:

(29) $$\begin{array}{l} \dot{V}=s^{T}\dot{s}+\frac{1}{\upsilon }\tilde{\varphi }\dot{\hat{\varphi }}\\ =s^{T}\left\{-hs-(\alpha +\hat{\varphi })sat(s)-\tilde{\lambda }\right\}-\tilde{\varphi }\left\|s\right\|\\ =-s^{T}hs-s^{T}\alpha sat(s)-s^{T}\hat{\varphi }sat(s)-s^{T}\tilde{\lambda }-\tilde{\varphi }\left\|s\right\|\\ \leq -s^{T}hs-s^{T}\alpha sat(s)-\left\|s\right\|(\hat{\varphi }-\varphi +\tilde{\varphi })\\ =-s^{T}hs-s^{T}\alpha sat(s) \end{array}$$

Now, inserting Equation (22) into Equation (29) yields:

(30) $$\begin{array}{l} \dot{V}\leq -s^{T}\alpha sat(s)-s^{T}hs\\ =\left\{ \begin{array}{l} -\sum_{i=1}^{7}\left(\frac{\alpha_{ii}{\left|s_{i}\right|}^{2}}{\delta }+h_{ii}{\left|s_{i}\right|}^{2}\right),\;\mathrm{when}\;\left|s\right|\leq \delta \\ -\sum_{i=1}^{7}\left(\alpha_{ii}\left|s_{i}\right|+h_{ii}{\left|s_{i}\right|}^{2}\right),\;\mathrm{when}\;\left|s\right|>\delta \end{array}\right.\\ \leq 0 \end{array}$$

Here, it can be seen that *V* is positive definite and $\dot{V}$ is negative definite. Besides, when ‖s‖ tends to infinity, *V* also tends to infinity. Therefore, the proposed control algorithm satisfies the Lyapunov stability criteria and, thus, is globally asymptotically stable. The position tracking error gradually converges to zero and approaches the sliding surface in finite time.

### 4.2. CPID-Based Impedance Control

Existing clinical stroke therapy experience shows that, for the stoke patients whose motion-relative central nervous systems have recovered to certain degree, integrating the motion intention and volunteer effort of patients into the rehabilitation training helps to improve treatment efficiency and regain self-confidence. During the patient-cooperative mode training, the patient need to sit on the wheelchair with his/her affected arm wearing the exoskeleton and palm grasping the end-effector. The relationship between robot configuration and human-robot interaction force at the end-effector can be modulated via an impedance controller.

The desired impedance property between exoskeleton and impaired arm can be defined as [43]:

(31) $$M_{d}\left({\ddot{x}}_{d}-\ddot{x}\right)+B_{d}\left({\dot{x}}_{d}-\dot{x}\right)+K_{d}\left(x_{d}-x\right)=F_{d}\;$$

where *M_d_*, *B_d_*, and *K_d_* denote the objective inertial matrix, damping matrix, and stiffness matrix of the impedance controller; ${\ddot{x}}_{d}$, ${\dot{x}}_{d}$, and $x_{d}$ represent the desired position, velocity, and acceleration of end-effector in Cartesian space; $\ddot{x}$, $\dot{x}$, and x represent the actual position, velocity, and acceleration of end-effector in Cartesian space; *F_d_* denotes the desired human-robot interaction force at the end-effector. It should be pointed out that the desired and actual position of end-effector can be calculated via the reference joint trajectory *θ_d_*, actual joint trajectory *θ*, and the forward kinematics of exoskeleton proposed in our previous research [44]. The corresponding velocity and acceleration can be obtained via deviation calculation. The desired human-robot interaction forces at the end-effector can be converted into the desired robot joint torques via the Jacobian matrixes as: $\tau_{d}=J_{2}^{T}F_{d}$

Then, an inner CPID force controller combined with a friction compensation module is integrated into the impedance controller to guarantee force control accuracy. The CPID control strategy can rapidly response to unknown disturbance and enhance force control performance. The parameters of the inner loop PID controller and outer loop PID controller are firstly estimated using the Ziegler-Nichols method [45] and optimally adjusted via intensive tests to reduce force control error and enhance overall system stability.

With the proposed CPID-based impedance controller, the disabled patients are allowed to actively adjust training trajectory and dominate the rehabilitation treatment process based on their motion intension. Furthermore, the virtual game therapy is integrated into the exoskeleton system to motivate patients with simple games presented on the audiovisual guidance interface running on host computer. The real-time information about exoskeleton configuration and human-robot interaction, which are acquired from force/torque sensors, position sensors, as well as the switches and pushbuttons mounted at the end-effector of exoskeleton, are transmitted into a virtual keyboard module to handle the virtual game process. The virtual keyboard module is developed in the Microsoft Visual C++ programming environment and used as the interface between the operator and virtual environment.

## 5. Experiments and Discussion

**Figure 6:**
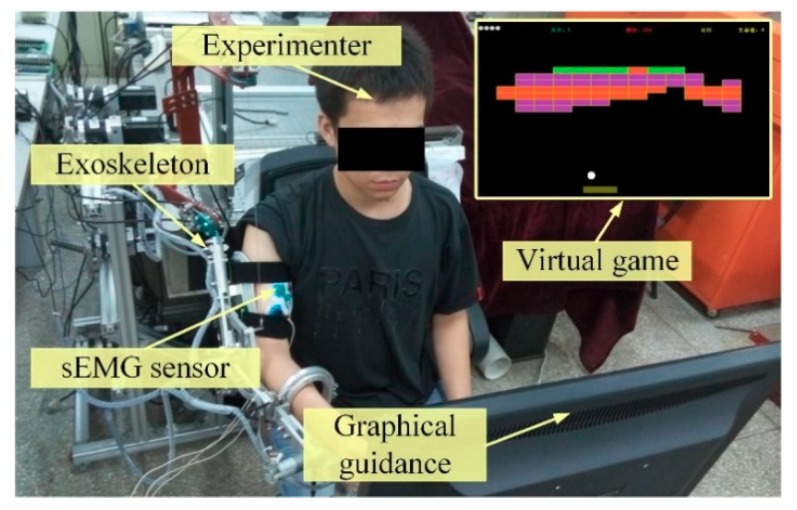
The upper-limb exoskeleton prototype worn by a volunteer looking at the graphical guidance.

With the aim of validate the feasibility and effectiveness of the proposed multi-modular control strategy, three representative experiments, including the trajectory-tracking experiments, the trajectory-tracking experiments with impedance adjustment, and the intention-based training experiments, were conducted by three volunteers with different anthropometric parameters (S1: male, weight/65 kg, height/1.71 m, age/30; S2: female, weight/52 kg, height/1.65 m, age/26; S3: male, weight/71 kg, height/1.79 m, age/46). Figure 6 shows the scenarios of a volunteer performing rehabilitation training under the graphical guidance shown screen. The ethical approval of the implemented experimental protocols has been obtained from the Institutional Review Board of the Nanjing University of Aeronautics and Astronautics.

### 5.1. Trajectory Tracking Experiments

For the fully paralyzed stroke patients remaining in the acute period without any muscle contraction, performing patient-passive repetitive training helps to motivate muscle contraction and avoid deterioration of motor function. The trajectory tracking experiments without active participation of wearer were carried out to evaluate the position control performance of the developed adaptive sliding mode controller with disturbance observer. During these experiments, the volunteers were comfortably equipped with the exoskeleton and commanded to perform right arm rehabilitation repetitive training on the shoulder flexion/extension joint, the elbow flexion/extension joint and the forearm pronation/supination movement. The state machine of the multi-modal controller shown in Figure 5 is switched to the patient-passive training mode. The feedback signals from the force/torque sensor and rotary potentiometers were detected and input into the adaptive sliding control strategy to provide effective motion assistance, as shown in the Figure 3. The desired trajectories of the selected three robotic joints are defined to simultaneously follow a sinusoid wave trajectory with a frequency of 0.25 Hz and a time-varying amplitude, while other robotic joints are limited to their initial positions. The duration of each experiment is set to 8 s. To facilitate the evaluation of the proposed control scheme, the control performance of the adaptive sliding mode controller with disturbance observer is compared to that of a conventional terminal sliding mode controller [46].

**Figure 7:**
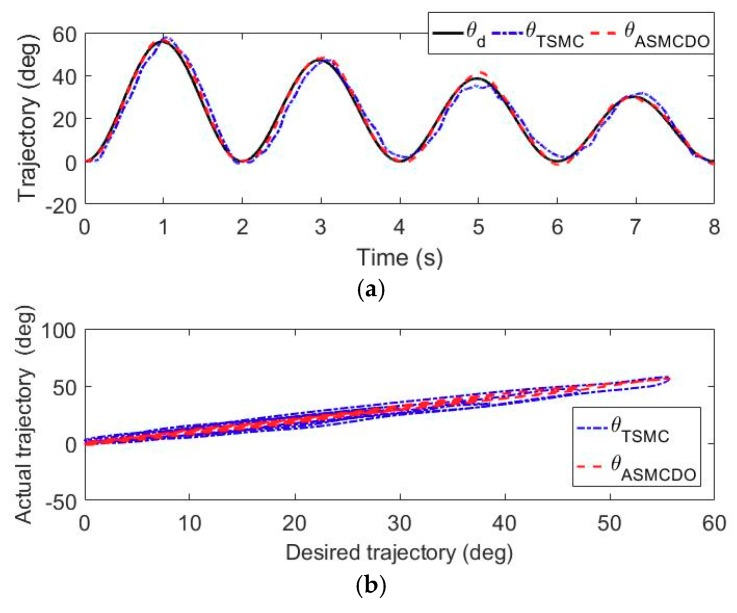

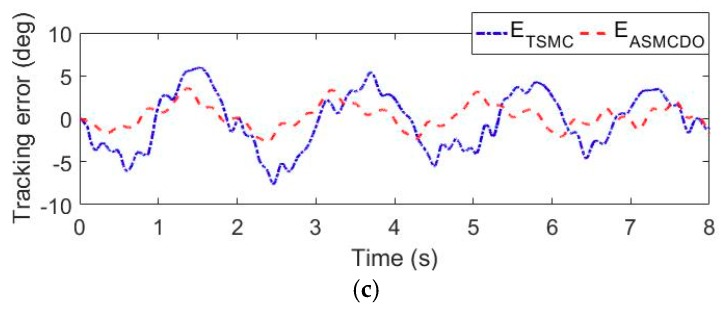
The comparison results of the trajectory tracking experiments for shoulder flexion/extension. (**a**) Time histories of desired displacement and actual displacement. (**b**) Comparison between desired displacement and actual displacement. (**c**) The tracking error of different controllers.

**Figure 8:**
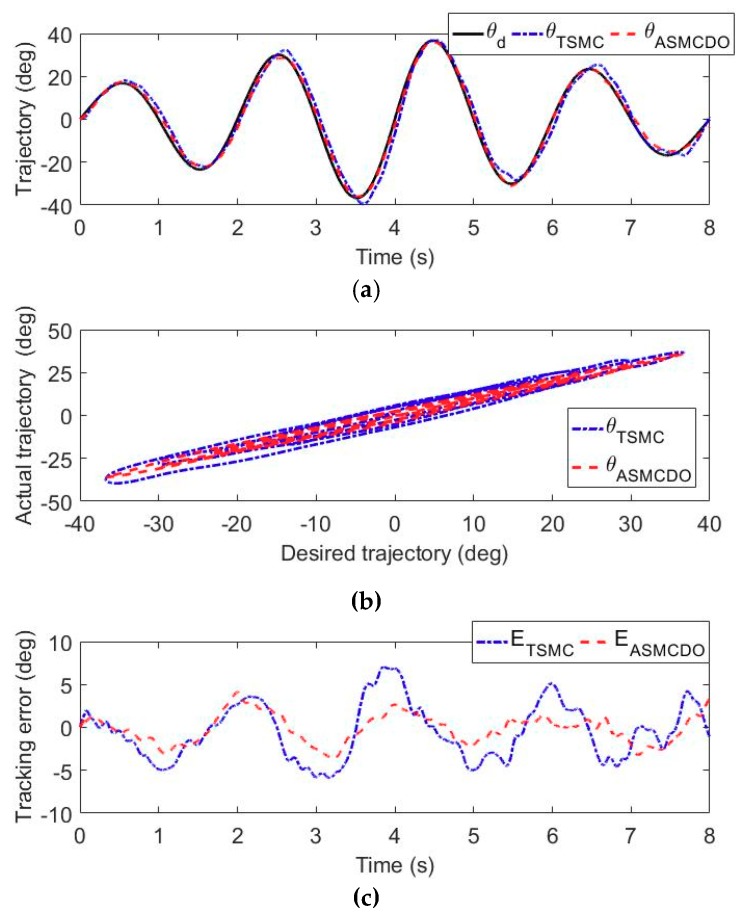
The comparison results of the trajectory tracking experiments for elbow flexion/extension. (**a**) Time histories of desired displacement and actual displacement. (**b**) Comparison between desired displacement and actual displacement. (**c**) The tracking error of different controllers.

**Figure 9:**
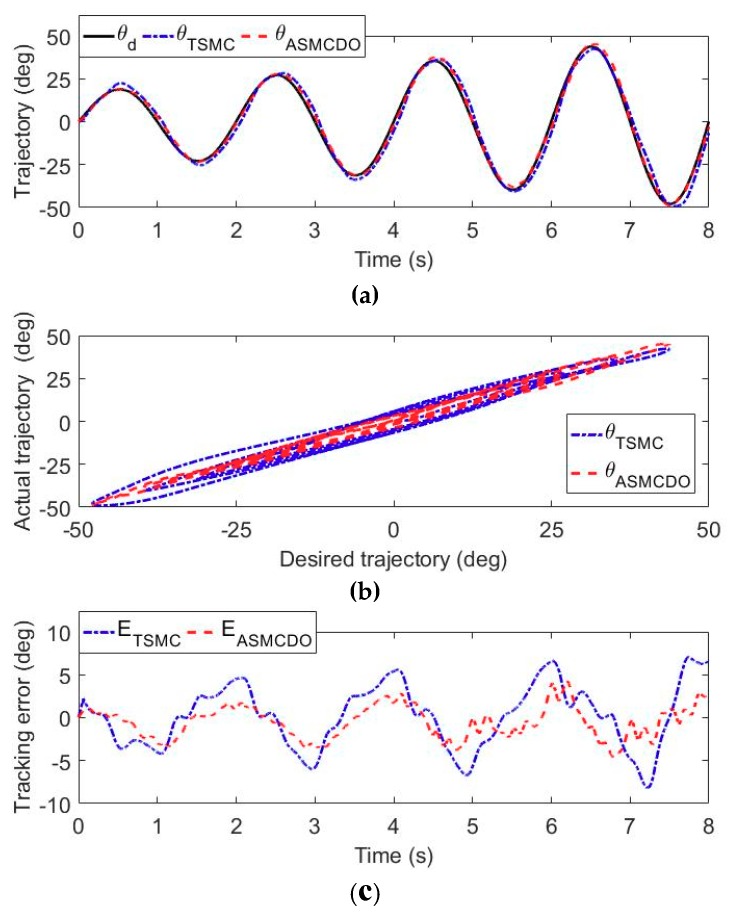
The comparison results of the trajectory tracking experiments for forearm pronation/supination movement. (**a**) Time histories of desired displacement and actual displacement. (**b**) Comparison between desired displacement and actual displacement. (**c**) The tracking error of different controllers.

The results of the trajectory tracking experiments carried out by the volunteer S1 are shown in Figure 7, Figure 8 and Figure 9. More specifically, the time histories of the desired trajectory and the actual trajectories under TSMC strategy and ASMCDO strategy are depicted in Figure 7a, Figure 8a and Figure 9a, respectively. The comparison between the desired and actual displacements are presented in Figure 7b, Figure 8b and Figure 9b respectively. Furthermore, the tracking errors of different control algorithms are compared in Figure 7c, Figure 8c and Figure 9c. It can be clearly found that the robot joints can follow the desired trajectories, and position control accuracy of the proposed ASMCDO control strategy is higher than that of the conventional TSMC strategy. For the purpose of further analyzing the position control performance with different control schemes, the maximum absolute error (MAXE) and mean absolute error (MAE) of the trajectory tracking results are defined as follows:

(32) $$MAXE=\max\left|e_{i}\right|\;$$

(33) $$MAE=\frac{1}{Q}\sum_{i=1}^{Q}\left|e_{i}\right|\;$$

where *e_i_* represents the *i*th error data; *Q* denotes the number of error data.

For the trajectory experiments of shoulder flexion/extension joint, it is seen that the proposed ASMCDO control strategy produces MAXE, RMSE and MAE of 3.56°, 1.42° and 1.16°, respectively. The conventional TSMC scheme leads to the MAXE of 7.61°, the RMSE of 3.37°, and the MAE of 2.88°. As compared with the TSMC, the ASMCDO strategy reduces MAXE, RMSE and MAE by 53.21%, 57.86%, and 59.72%, respectively. For the trajectory tracking experiments of elbow flexion/extension joint, MAXE declines from 7.09° (TSMC) to 4.11° (ASMCDO), the RMSE declines from 3.30° (TSMC) to 1.72° (ASMCDO), and the MAE declines from 2.81° (TSMC) to 1.43° (ASMCDO). The ASMCDO strategy reduces MAXE, RMSE and MAE by 42.03%, 47.87%, and 49.11%. Regarding the trajectory tracking experiments of forearm pronation/supination, the MAXE declines from 8.17° (TSMC) to 4.60° (ASMCDO), the RMSE declines from 3.63° (TSMC) to 1.96° (ASMCDO), and the MAE declines from 2.99° (TSMC) to 1.61° (ASMCDO). The ASMCDO strategy reduces MAXE, RMSE and MAE by 43.69%, 46.01%, and 46.16%. The errors of TSMC scheme are larger than the those of ASMCDO control strategy. Moreover, the detailed statistical results from the trajectory tracking experiments conducted by S1, S2 and S3 are all tabulated in Table 3. Clearly, it can be concluded that the developed ASMCDO control strategy is effective in reducing the position tracking error and guaranteeing the control performance during patient-passive repetitive rehabilitation training. The experimental results reveal the superiority of the proposed ASMCDO controller over the traditional TSMC scheme in terms of position control precision.

### 5.2. Trajectory Tracking Experiments with Impedance Adjustment

**Figure 10:**
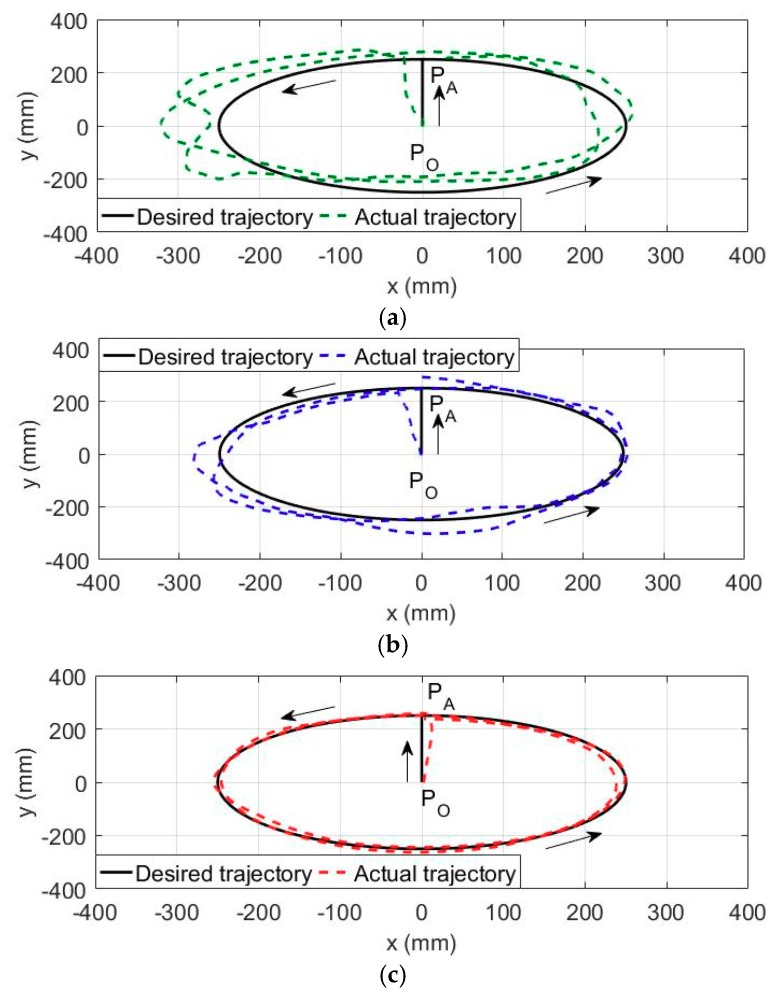
The results of the trajectory tracking experiments with impedance adjustment conducted by S1. (**a**) Low impedance parameters. (**b**) Medium impedance parameters. (**c**) Large impedance parameters.

The purpose of the aforementioned patient-passive mode training is to guarantee the position control accuracy without participation of wearer. However, for the patients regaining parts of motor functions, the repetitive passive training with impedance adjustment, which is able to regulate the training trajectory based on real-time human-robot interaction force, helps to improve the compliance, comfort and safety during rehabilitation training. In these experiments, the state machine of the multi-modal controller is switched to the patient-cooperative training mode. The volunteers were required to wear the therapeutic exoskeleton on their right arm and grasp the end-effector. The desired trajectory of end-effector is defined to follow a circular path parallel to the coronal plane in the base robot coordinate frame of exoskeleton, as shown in Figure 10. The initial configuration of the exoskeleton is depicted in Figure 1, and the initial location of end-effector is set to point P_o_. And then, the end-effector is controlled to move horizontally from point P_o_ to point P_A_ in two seconds. Point P_o_ is defined as the center of circular path, and the radius of the circular path, i.e., |P_A_P_o_|, is set to 250 mm. After that, the end-effector is controlled to follow the circular path in counterclockwise direction for two cycles, and the cycle time is set to 10 s. The desired trajectory of end-effector can be converted to the trajectory of each robot joint through the inverse kinematic model. The proposed CPID-based impedance controller is capable of adjusting the training trajectory based on the interaction force detected by force/torque sensor and the predefined impedance parameters. With the aim of analyzing the control performance of the developed CPID-based impedance controller, the volunteers are commanded to perform the circular trajectory tracking experiments with three groups of different impedance parameters (i.e., *M_d_*, *B_d_*, and *K_d_*), including low impedance parameters, medium impedance parameters, and large impedance parameters, as shown in Table 4. 

Figure 10 presents the experimental results conducted by S1. The trajectory tracking experiment with low impedance adjustment produces MAXE, RMSE and MAE of 82.79 mm, 44.23 mm and 39.71 mm, respectively. The trajectory tracking experiment with medium impedance adjustment produces MAXE, RMSE and MAE of 51.75 mm, 29.01 mm and 25.36 mm, respectively. The trajectory tracking experiment with large impedance adjustment produces MAXE, RMSE and MAE of 16.64 mm, 10.41 mm and 9.57 mm, respectively. Overall, as compared with results in low impedance condition, the MAXE, RMSE and MAE of medium impedance experiments are reduced by 37.49%, 34.41%, and 36.13%, while the MAXE, RMSE and MAE of large impedance experiments are reduced by 79.90%, 78.36%, and 75.91%, respectively. 

Furthermore, the detailed statistical results from the trajectory tracking experiments with impedance adjustment conducted by S1, S2 and S3 are all tabulated in Table 4. It can be observed from the experimental results that the deviation between the desired and actual training trajectories tends to decrease with the increase of impedance parameters. Therefore, by rationally adjusting the impedance parameters of the proposed CPID-based impedance controller, the compliance of human-robot interaction can be modulate to meet the passive-training requirement of the patients with various weakness levels.

### 5.3. Intention-Based Training Experiments

For the patients regaining most of motor functions at the recovery stage, the rehabilitation training integrated with the active intention and volunteer effort of patients contributes to recover psychological confidence and improve therapy effectiveness. Thus, the intention-based training experiments were carried out by three volunteers to verify the feasibility of the proposed CPID-based impedance control strategy in providing intention-based patient-cooperation training. In these experiments, the state machine of the multi-modal controller is switched to the patient-cooperative training mode, and the desired trajectory of end-effector is fixed at the point P_o_. Firstly, the volunteers were required to wear the exoskeleton with their right arms and grasp the end-effector. The position of end-effector along the horizontal x-direction of the base robot coordinate frame is mapped into the position of moving flapper in the virtual game. Then, the volunteers need to actively manipulate the exoskeleton to conduct horizontal reciprocating training and control the motion of virtual flapper with the audiovisual guidance displayed on screen. In the virtual game, the moving flapper needs to be controlled to hit the falling marble and remove the obstacle located at the upside of screen. According to Equation (31), the interaction force applied by the wearer along the horizontal x-direction lead to the position deviation of end-effector in the same direction. The position deviation of end-effector is basically limited in the range of [−300 mm, 300 mm], which satisfies the valid range of motion of flapper in virtual environment. The relationship between interaction force and position deviation can reflect the training intensity during intention-based training. For the similar training task, the larger the interaction force applied by the wearer, the greater the training intensity. With the aim of analyzing the training effect with different impedance parameters, three groups of impedance parameters, as shown in Table 4, were used in the intention-based experiments. Each volunteer performed the repetitive moving task five times for each impedance condition, and the duration of each test lasted for about 15 s. The existing investigations results reveal that the strength of surface electromyography (sEMG) signals reflect the scale and variation of human limb muscular power. Therefore, the sEMG signals from the bicipital muscle and muscle of the wearer were measured by a high-precision sEMG signal measurement system (Sichiray, Sunlephant-6 sEMG System, Shenzhen, China), with the aim of qualitatively evaluating the muscular power during the rehabilitation training with different impedance parameters.

**Figure 11:**
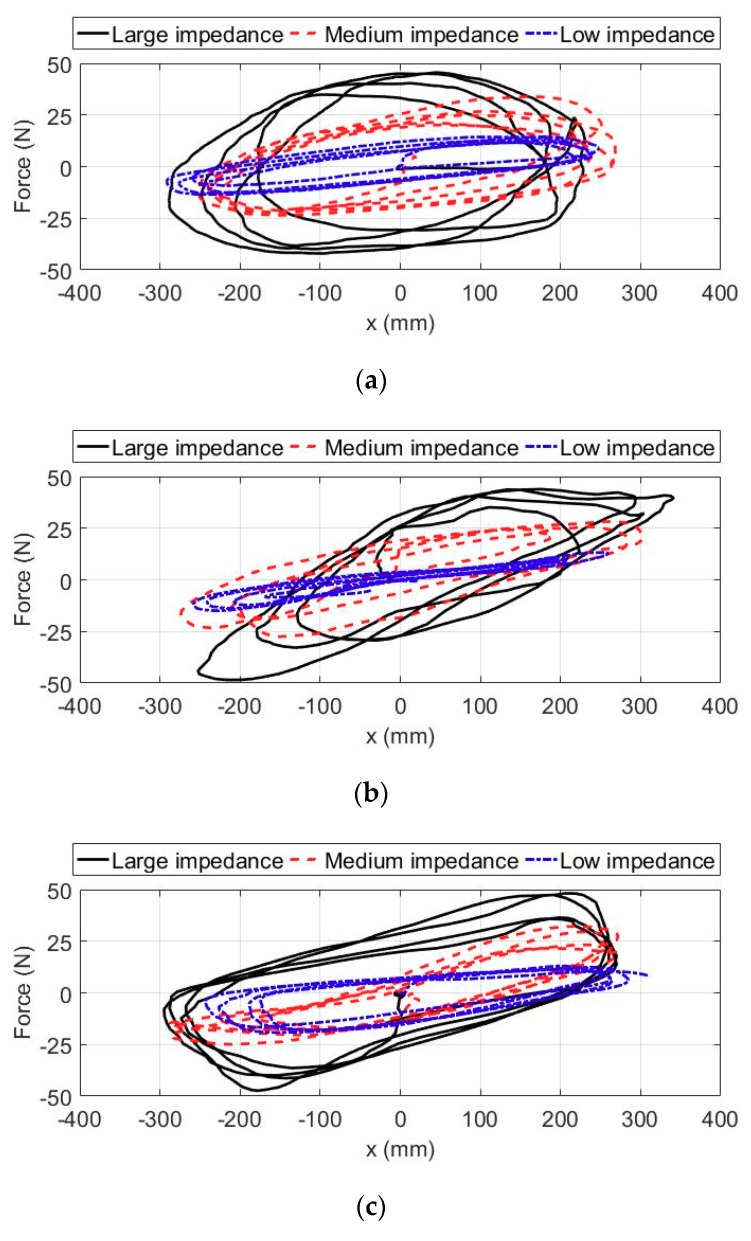
The results of the intention-based experiments with different impedance parameters and different volunteers in a single trial. (**a**) The experimental results of S1. (**b**) The experimental results of S2. (**c**) The experimental results of S3.

The results of the intention-based experiments with different impedance parameters and different volunteers in a single trial are compared and shown in Figure 11. The volunteers equipped with the exoskeleton are all capable of actively handling the movement of flapper in virtual game during these experiments. It can be seen from the experimental results that the position deviation of end-effector along the horizontal x-direction is basically consistent with the value of the human-robot interaction force. Besides, the increase of impedance parameters leads to larger interaction force. The interaction forces with large impedance parameters, medium impedance parameters, and low impedance parameters are basically limited within the ranges of [−50 N, 50 N], [−25 N, 25 N], and [−15 N, 15 N], respectively. In this way, the disabled patients are allowed to actively manipulate the therapeutic exoskeleton based on their subjective intention and perform rehabilitation training under graphical feedback guidance. The sEMG signals from the bicipital and triceps muscles are added and featured by utilizing the root mean square (RMS) methods. 

**Figure 12:**
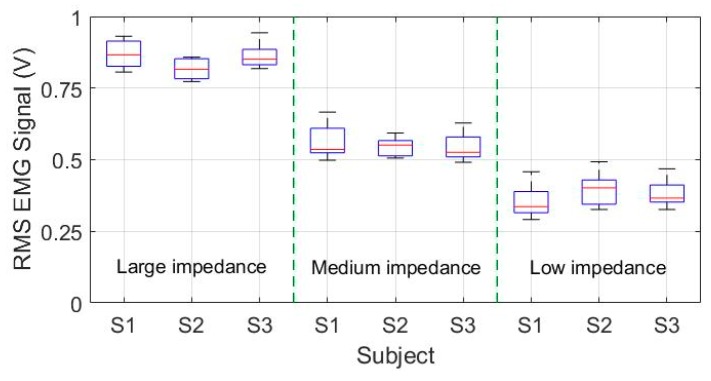
The comparison results of the RMS EMG values of different subjects and impedance parameters in five trials.

Figure 12 and Table 5 compare the experimental results of the RMS sEMG values of different volunteers and different impedance parameters in five trials. The median values in the large impedance condition are about 0.856 V (S1), 0.815 V (S2) and 0.849 V (S3), respectively. In comparison, the median values in the medium impedance condition are about 0.535 V (S1), 0.551 V (S2) and 0.525 V (S3). The median values in the low impedance condition are about 0.336 V (S1), 0.402 V (S2) and 0.369 V (S3). The statistical results show that the RMS sEMG values under the same experimental condition vary from person to person and trial to trial. However, in general the sEMG activity levels of the experiments with large impedance parameters are greater than those with medium small impedance parameters and small impedance parameters. The decrease of impedance parameters lead to the increase of human limb muscular power during rehabilitation training. Therefore, it can be concluded from the results that the motion resistance and training intensity during intention-based training can be adjusted via the impedance parameters, and it facilitates to meet the training requirements of patients with different hemiplegia degrees and therapy programs.

## 6. Conclusions

The feasibility of a therapeutic upper limb exoskeleton and a multi-modal control strategy for stroke patients with different degrees of hemiplegia is investigated in this research. The dynamic model of the human-robot interaction system is established, and the dynamics parameters are identified via computer-aided virtual prototype and experiments. The multi-modal control strategy is composed of an adaptive sliding mode controller with disturbance observer (ASMCDO) to provide patient-passive training and a cascade-proportional-integral-derivative (CPID)-based impedance controller to provide patient-cooperative training. The effectiveness of the developed exoskeleton system and control scheme are validated via three experiments, including the trajectory tracking experiments, the trajectory tracking experiments with impedance adjustment, and the intention-based experiments with virtual graphical guidance. The experimental results indicate that the position control accuracy of patient-passive training can be guarantee via the ASMCDO controller. Besides, the proposed CPID-based impedance control strategy is capable of improving the interaction compliance and activating active participation of patients during rehabilitation training. Moreover, the training difficulty and intensity can be rationally adjust to satisfy the requirements of patients with difficult weakness levels. Future works will be devoted to evaluating the effectiveness of the proposed robot-assisted therapeutic system in clinical application. The training trajectory for specific clinical application will be studied. Besides, the optimization of impedance parameters will investigated by integrating with the clinical experience of physiotherapists to further improve the rehabilitation efficiency and effectiveness.

## Figures and Tables

**Table 1:** Kinematics and dynamics parameters of the upper limb exoskeleton

Link *i*	*θ_i_*/Home (deg)	Mass (kg)	Link Length (mm)	Inertia (kg·m^2^·10^−3^)	ROM (deg)
1	*θ*_1_/180	1.48	300	58.1	150~240
2	*θ*_2_/−60	0.71	185	16.6	−180~−45
3	*θ*_3_/−90	1.25	320~380	75.9~77.6	−120~30
4	*θ*_4_/−90	0.79	156	13.5	−180~−30
5	*θ*_5_/0	0.31	108.5~148.5	2.26~2.45	−85~60
6	*θ*_6_/90	0.09	100	0.72	80~120
7	*θ*_7_/0	0.42	95.5	1.63	−30~60

**Table 2:** Identification results for the parameters of Coulomb-viscous friction models.

Joint	Coulomb Coefficient (N·m)	Viscous Coefficient (N·m·s/deg)	RMSE (N·m)
Shoulder internal/external	2.81	0.041	0.32
Shoulder abduction/adduction	4.95	0.021	0.78
Shoulder flexion/extension	3.86	0.035	0.62
Elbow flexion/extension	4.10	0.020	0.42
Forearm pronation/supination	2.52	0.018	0.49
Wrist flexion/extension	0.21	0.002	-
Wrist ulnal/radial deviation	0.21	0.002	-

**Table 3:** Statistical results of the trajectory tracking experiments with different control strategies and different volunteers.

Subject	Controller	Shoulder Flexion/Extension			Elbow Flexion/Extension			Forearm Pronation/Supination		
		MAXE (°)	RMSE (°)	MAE (°)	MAXE (°)	RMSE (°)	MAE (°)	MAXE (°)	RMSE (°)	MAE (°)
S1	TSMC	7.61	3.37	2.88	7.09	3.30	2.81	8.17	3.63	2.99
	ASMCDO	3.56	1.42	1.16	4.11	1.72	1.43	4.60	1.96	1.61
S2	TSMC	7.82	3.41	2.95	7.57	3.83	3.01	8.23	3.69	3.23
	ASMCDO	3.65	1.56	1.33	4.35	2.10	1.69	4.72	2.07	1.72
S3	TSMC	8.05	3.92	3.02	7.63	3.79	3.22	8.61	3.87	3.36
	ASMCDO	3.74	1.59	1.37	4.58	2.23	1.81	4.83	2.23	1.79

**Table 4:** The results of trajectory tracking experiments with impedance adjustment under different experimental conditions (i.e., different impedance parameters).

Experimental Condition	Impedance Parameters			Subject	MAXE (mm)	RMSE (mm)	MAE (mm)
	*M_d_* (N·s^2^/mm)	*B_d_* (N·s/mm)	*K_d_* (N/mm)				
Low impedance	diag [0.015, 0.015, 0.015]	diag [0.015, 0.015, 0.015]	diag [0.015, 0.015, 0.015]	S1	82.79	44.23	39.71
				S2	88.65	46.96	41.77
				S3	87.34	47.03	43.31
Medium impedance	diag [0.035, 0.035, 0.035]	diag [0.035, 0.035, 0.035]	diag [0.035, 0.035, 0.035]	S1	51.75	29.01	25.36
				S2	49.22	28.34	23.39
				S3	53.98	31.73	27.50
Large impedance	diag [0.065, 0.065, 0.065]	diag [0.065, 0.065, 0.065]	diag [0.065, 0.065, 0.065]	S1	16.64	10.41	9.57
				S2	17.76	12.34	10.69
				S3	18.05	14.25	11.43

**Table 5:** The statistical results of the RMS sEMG values of bicipital and triceps muscles of different volunteers under different impedance parameters.

Experimental Condition	RMS EMG Values of Different Subjects (V)								
	S1			S2			S3		
	Max ^1^	Med ^2^	Min ^3^	Max	Med	Min	Max	Med	Min
Large impedance	0.931	0.856	0.803	0.859	0.815	0.773	0.943	0.849	0.818
Medium impedance	0.663	0.535	0.498	0.593	0.551	0.506	0.628	0.525	0.493
Low impedance	0.457	0.336	0.291	0.497	0.402	0.323	0.467	0.369	0.325

^1^ Max = Maximum; ^2^ Med = Median; ^3^ Min = Minimum.
